# Supplementary material for: ERβ Regulation of Indian Hedgehog Expression in the First Wave of Ovarian Follicles
Source: Cells. 2024 Apr 6;13(7):644. doi: 10.3390/cells13070644 (PMC11011683; doi:10.3390/cells13070644)
Supplement: Supplementary file 1 [file cells-13-00644-s001.zip › cells-2821108-supplementary.pdf]

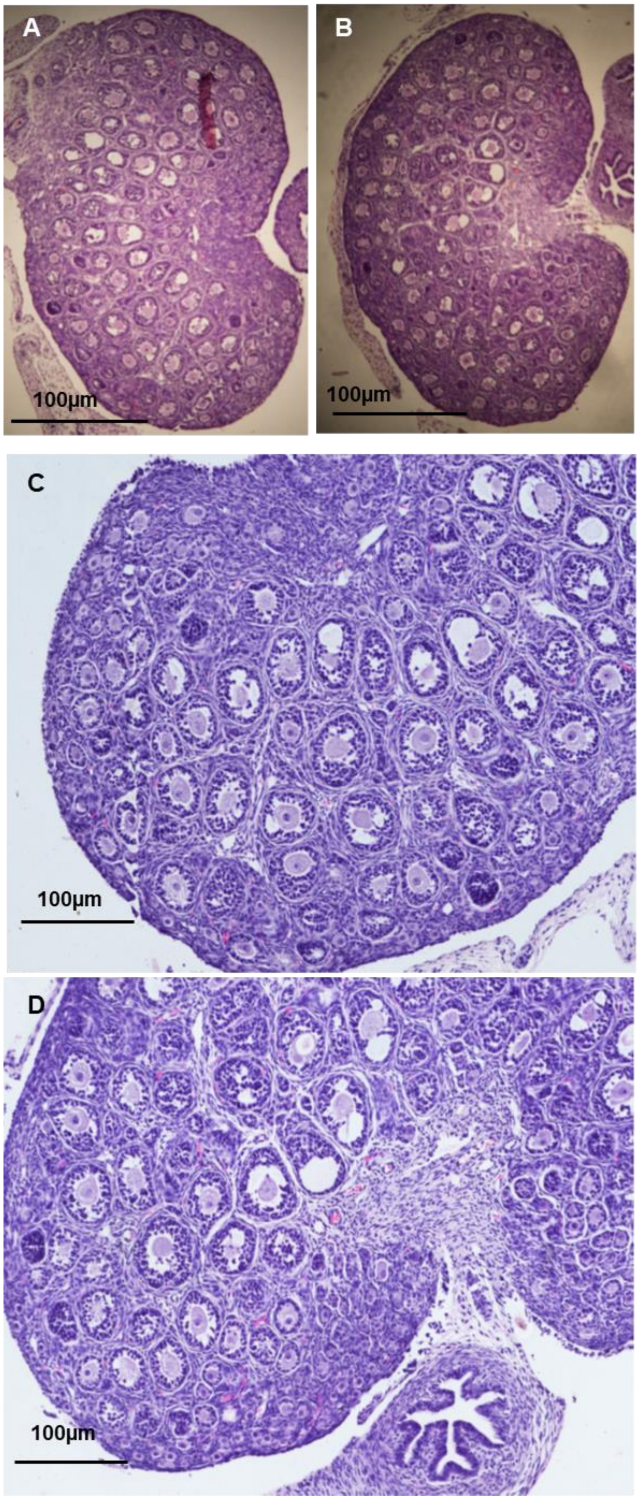

Supplementary Figure S1. Hematoxylin and Eosin stained histological sections of postnatal day 8.5 *Erβ*^{KO} rat ovaries. Image slides **A** and **B** are showing low magnification (10X) images of whole ovaries of rat 8.15 and 8.16. Image slides **C** and **D** are showing higher magnification of the part of the same two ovaries from rat 8.15 and 8.16. The images clearly showing that the larger follicles are not antral follicles, they are multilayered secondary follicles. Primordial follicles are seen in the cortical regions.

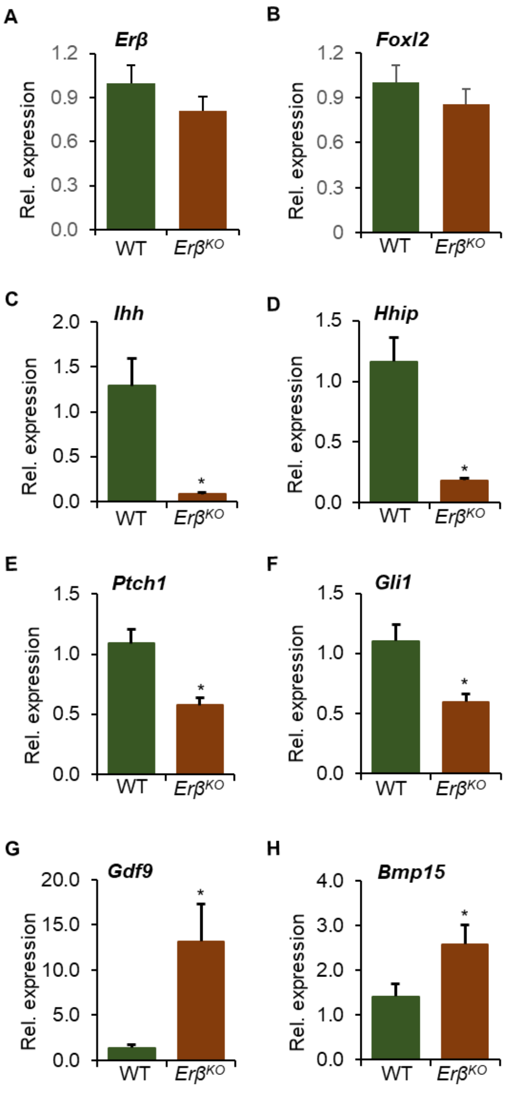

Supplementary Figure S2. Verification of hedgehog signaling genes. RNA-Seq data were validated using RT-qPCR analyses. cDNAs were prepared from total RNAs extracted from the PND 8.5 $Er\beta^{KO}$ and age-matched wildtype (WT) rat ovaries. RT-qPCR confirmed significant downregulation of *Erβ*, *Foxl2*, *Ihh*, *Hhip*, *Ptch1*, and *Gli1* genes (A-D), despite the marked upregulation of *Gdf9* and *Bmp15* (E, F). RT-qPCR data are shown as mean ± SE. $*p < 0.05$, n = 6.

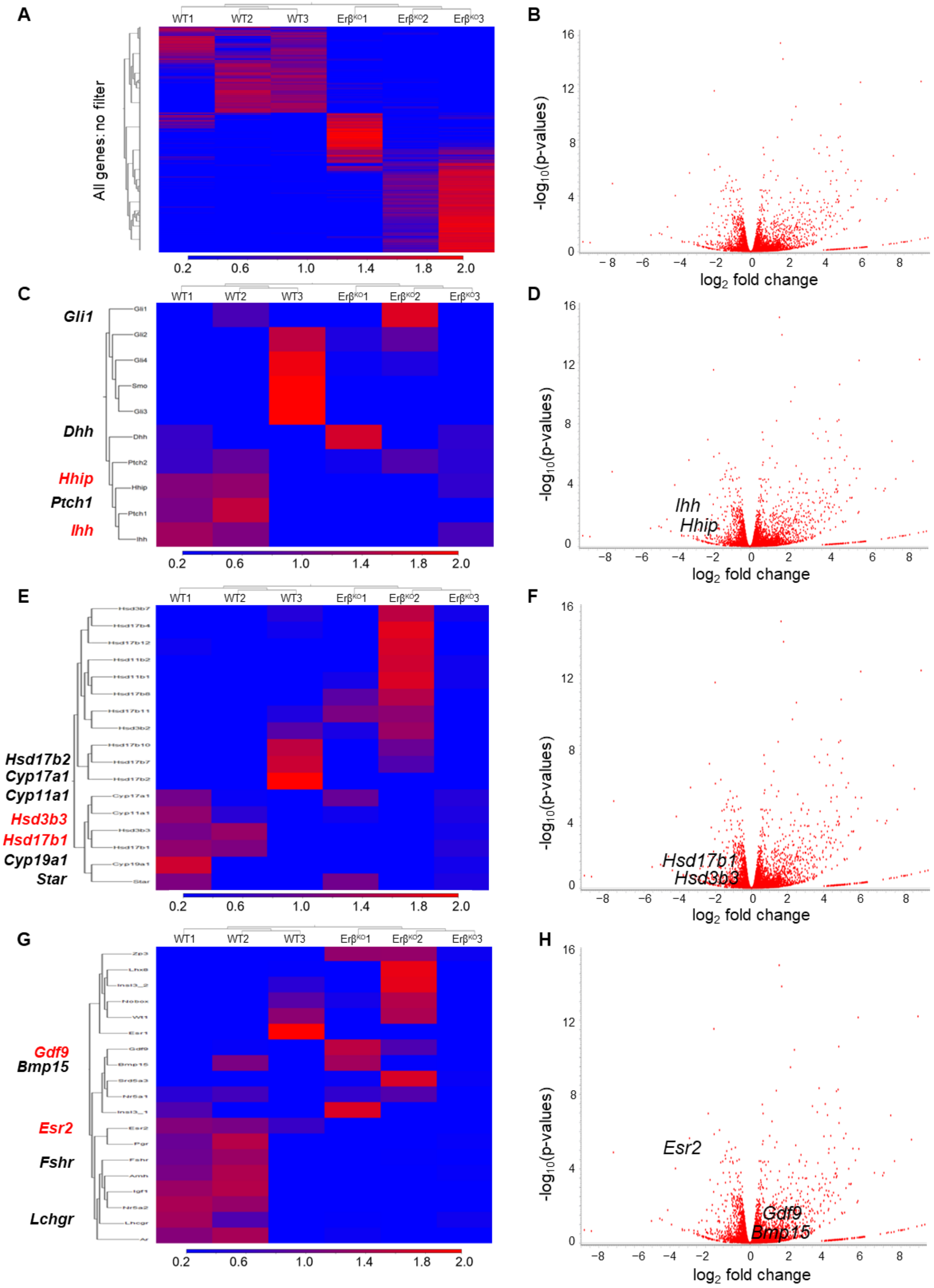

Supplementary Figure S3. RNA-Seq analysis of postnatal day 4.5 rat ovaries. RNA-Seq analysis (Heat maps in the left panel and Volcano plots in the right panel) of postnatal day 4.5 rat ovaries showing differential expression of genes in the whole ovary (no filter) (**A, B**), related to the hedgehog pathway (**C, D**), steroidogenesis (**E, F**), and the key genes involved in the process of folliculogenesis (**G, H**). Data are shown as CLC Genomic Workbench Analysis.

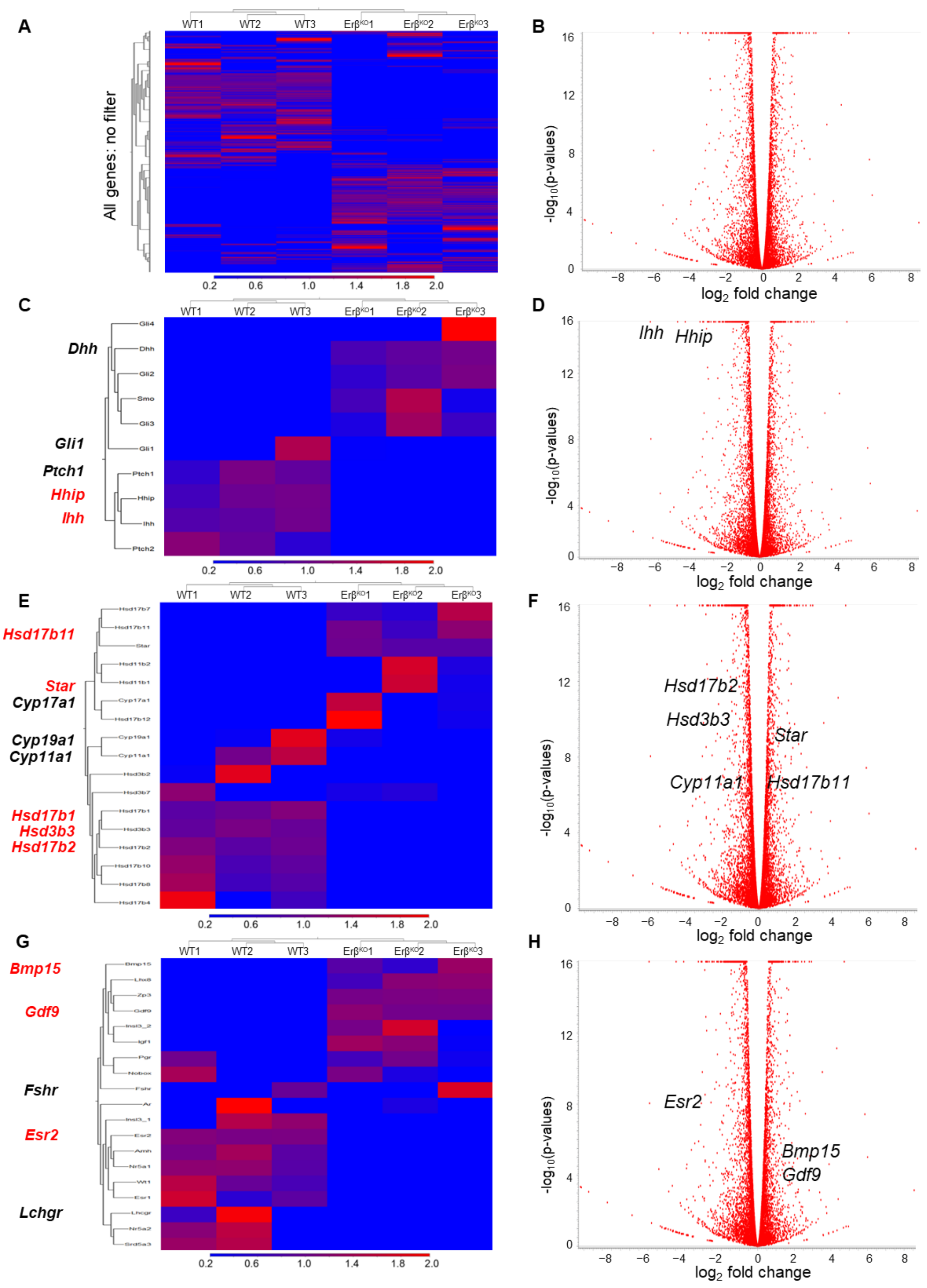

Supplementary Figure S4. RNA-Seq analysis of postnatal day 6.5 rat ovaries. RNA-Seq analysis (Heat maps in the left panel and Volcano plots in the right panel) of postnatal day 6.5 rat ovaries showing differential expression of genes in the whole ovary (no filter) (**A, B**), related to the hedgehog pathway (**C, D**), steroidogenesis (**E, F**), and the key genes involved in the process of folliculogenesis (**G, H**). Data are shown as CLC Genomic Workbench Analysis

A. Gene ontology

Panther GO-Slim Molecular Function

Differentially expressed genes in postnatal 8.5 $Er\beta^{KO}$ ovaries: 295

Total number of genes: 218. Total number of function hits: 273

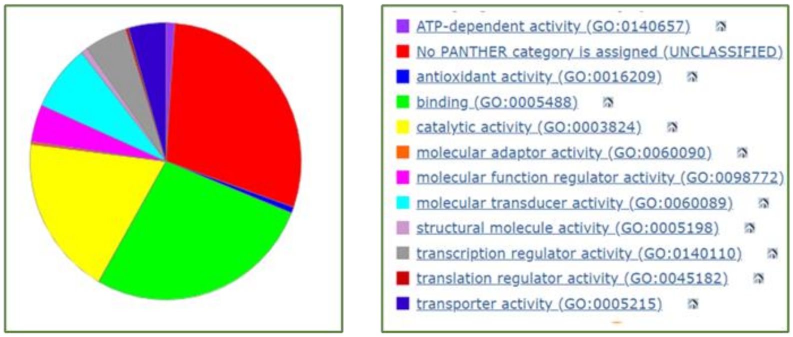

B. Graphical Summary

Differentially expressed genes in postnatal 8.5 $Er\beta^{KO}$ ovaries

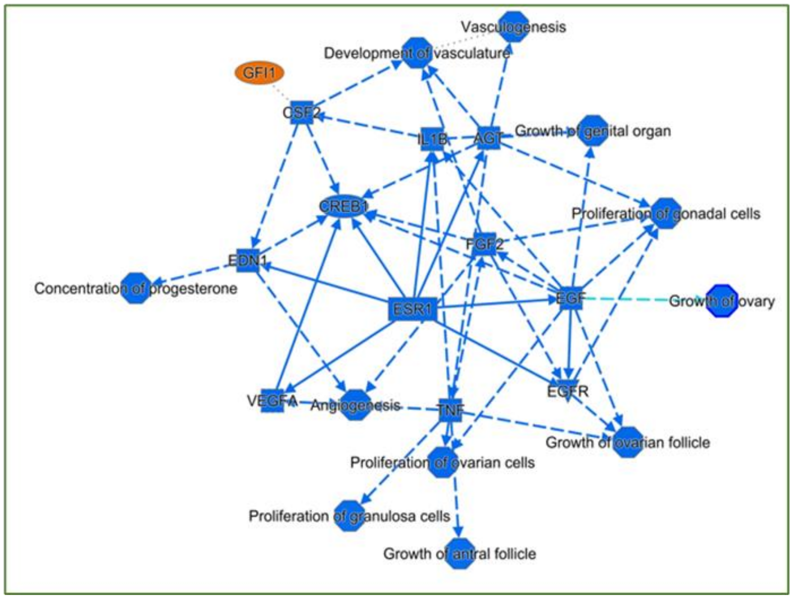

Supplementary Figure S5. Gene Ontology and IPA analysis of differentially expressed genes in $Er\beta^{KO}$ rat ovaries. Differentially expressed genes from postnatal day $Er\beta^{KO}$ and age-matched wildtype rat ovaries were subjected to Gene Ontology analysis that identified various molecular and functional pathways related to ATP-dependent activity, antioxidant activity, molecular adaptor activity, transporter activity, transcriptional and translational regulatory activity (**A**). When the DEGs were subjected to Ingenuity Pathway analysis, they revealed the upstream, and downstream signaling pathways, and involved physiological functions such as ovarian follicular development, ovarian granulosa cell development, and growth of ovarian follicles (**B**).

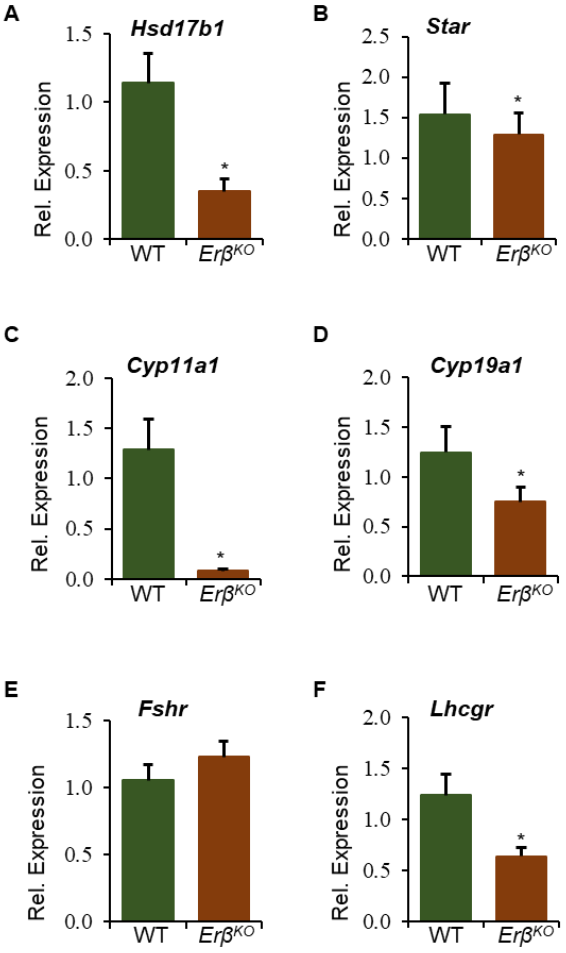

Supplementary Figure S6. Verification of the expression of steroidogenic genes. RNA-Seq data were validated using RT-qPCR. cDNAs were prepared from total RNAs extracted from the PND 8.5 $Er\beta^{KO}$ and age-matched wildtype (WT) rat ovaries. RT-qPCR analysis showed that the expression of *Hsd17b1*, *Cyp11a1*, and *Cyp19a1* steroidogenic enzymes were significantly downregulated. The expression of the gonadotropin receptor *Fshr* and *Lhcgr* also matched with the RNA-Seq results. RT-qPCR data are shown as mean ± SE. *$p < 0.05$, $n = 6$.
